# A Case of Drug-Induced Interstitial Lung Disease Caused by Epirubicin and Cyclophosphamide Therapy Before Breast Cancer Surgery

**DOI:** 10.7759/cureus.20676

**Published:** 2021-12-25

**Authors:** Ayaka Egashira, Toyoshi Yanagihara, Naruhiko Ogo, Tatsuma Asoh, Takashige Maeyama

**Affiliations:** 1 Department of Respiratory Medicine, Hamanomachi Hospital, Fukuoka, JPN

**Keywords:** interstitial lung disease, breast cancer, cyclophosphamide, epirubicin, drug-induced pneumonitis

## Abstract

We report a case of drug-induced interstitial lung disease (ILD) caused by epirubicin and cyclophosphamide (EC) therapy in a patient with breast cancer. The patient suffered from a dry cough, fever, and exertional dyspnea after two courses of EC therapy. Antibiotic treatment did not improve her symptoms. Chest CT images revealed diffuse, ground-glass opacities and mild interlobular septal thickening in both lungs, a pattern suggesting a hypersensitivity pneumonitis. Bronchoalveolar lavage fluid analysis revealed lymphocytosis with no evidence of infection nor malignancy. Corticosteroid therapy was initiated, which led to a rapid resolution of ILD. To date, there has been only one case report regarding drug-induced ILD caused by EC therapy. This case report could increase awareness of chemotherapy-induced pneumonitis.

## Introduction

Globally, breast cancer is the most common cancer in women [1], and systemic therapy is an essential part of the treatment for breast cancer, including endocrine therapy, targeted therapy, and chemotherapy, based on ﻿the three major breast cancer subtypes: hormone receptor positive/estrogen or progesterone receptors and human epidermal growth factor 2 (ERBB2) negative (HR+/ERBB2−), ERBB2 positive (ERBB2+), and triple negative [2]. With regard to chemotherapy, anthracycline-based regimens are the recommended therapies [2]. These systemic therapies can reduce the relapse rate and improve the prognosis of breast cancer. Nevertheless, they are toxic, and drug-induced interstitial lung disease (ILD) is one of the adverse events. Here, we report a case of drug-induced ILD caused by epirubicin and cyclophosphamide (EC) therapy before breast cancer surgery.

## Case presentation

A 55-year-old Japanese woman was referred to a breast surgery clinic after an abnormality was noted during a breast cancer screening five months prior. A 20-mm painless mass in the right D region was noted. Close examination resulted in a diagnosis of right breast cancer T1cN0M0, clinical Stage I, triple negative. EC therapy was initiated as a preoperative chemotherapy three months before. The mammary mass was reduced in size, but fever and dry cough appeared after two courses of EC administration. She was a nonsmoker. She was taking famotidine, magnesium oxide, and a prophylactic dose of sulfamethoxazole-trimethoprim. She was admitted to a local hospital. Antibiotic treatment (levofloxacin 500 mg/day, followed by cefepime 4 g/day) did not improve her symptoms. A PCR test for severe acute respiratory syndrome coronavirus 2 (SARS-CoV2) was negative. Blood cultures before starting antibiotics were negative. The patient had dry cough, exacerbation of respiratory distress, and mild hypoxia (percutaneous oxygen saturation (SpO_2_): around 90% in the supine position) and was transferred to Hamanomachi Hospital.

**Figure 1:**
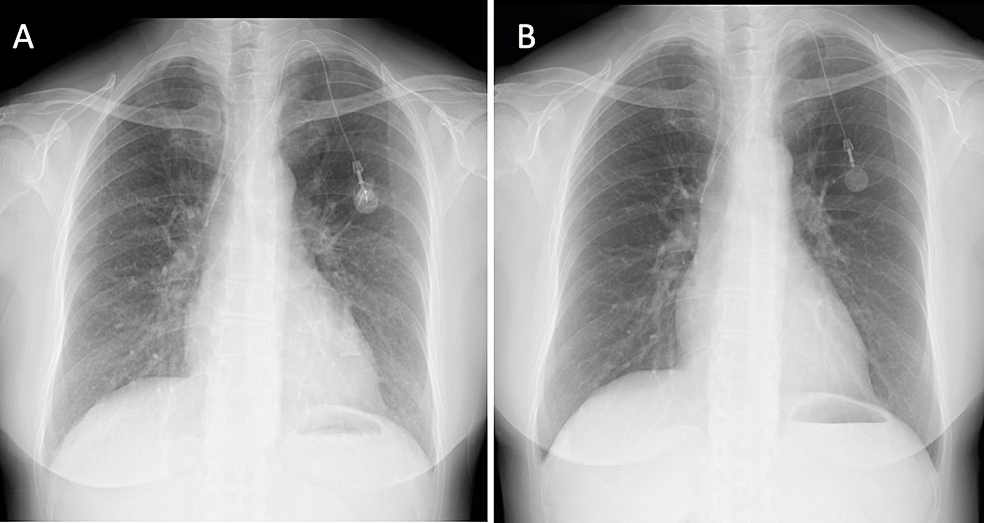
Chest radiographic images of the patient. Chest X-ray images (A) on admission and (B) at discharge.

Upon admission to Hamanomachi Hospital, the patient had a temperature of 36.4℃, blood pressure of 96/68 mmHg, and a heart rate of 113 beats per minute. Physical examination revealed fine crackles at her back. Her levels of C-reactive protein (CRP) and lactate dehydrogenase (LDH) were elevated at 3.77 mg/dL and 348 U/L, respectively. White blood cell count (WBC) was 3,600/µL (neutrophils: 81.1%, lymphocytes: 9.1%). Her level of Krebs von den Lungen-6 (KL-6) was within the normal range (320 U/mL; normal range <500 U/mL). Serum β-D glucan and antineutrophil cytoplasmic antibodies (ANCA) were negative. A chest X-ray image revealed a ground-glass appearance in both lung fields (Figures 1A, 1B).

**Figure 2:**
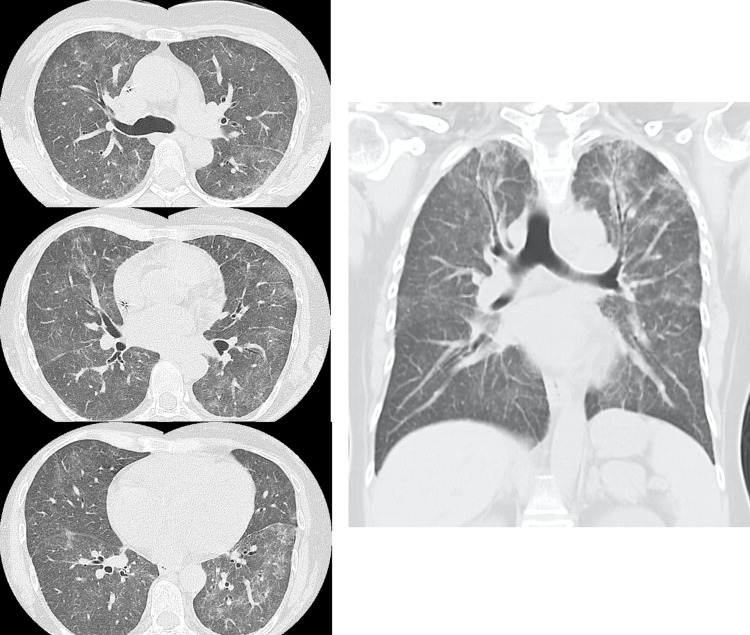
Chest CT images of the patient upon admission to Hamanomachi Hospital.

A CT scan of the chest showed diffuse ground-glass opacities and mild interlobular septal thickening in both lungs, predominantly in the upper lobes (Figure 2). No mediastinal lymphadenopathy nor traction bronchiectasis was observed. These CT findings were compatible with the hypersensitivity pneumonitis pattern according to the American Thoracic Society/Japanese Respiratory Society/Latin American Thoracic Society (ATS/JRS/ALAT) 2020 guideline [3].

**Figure 3:**
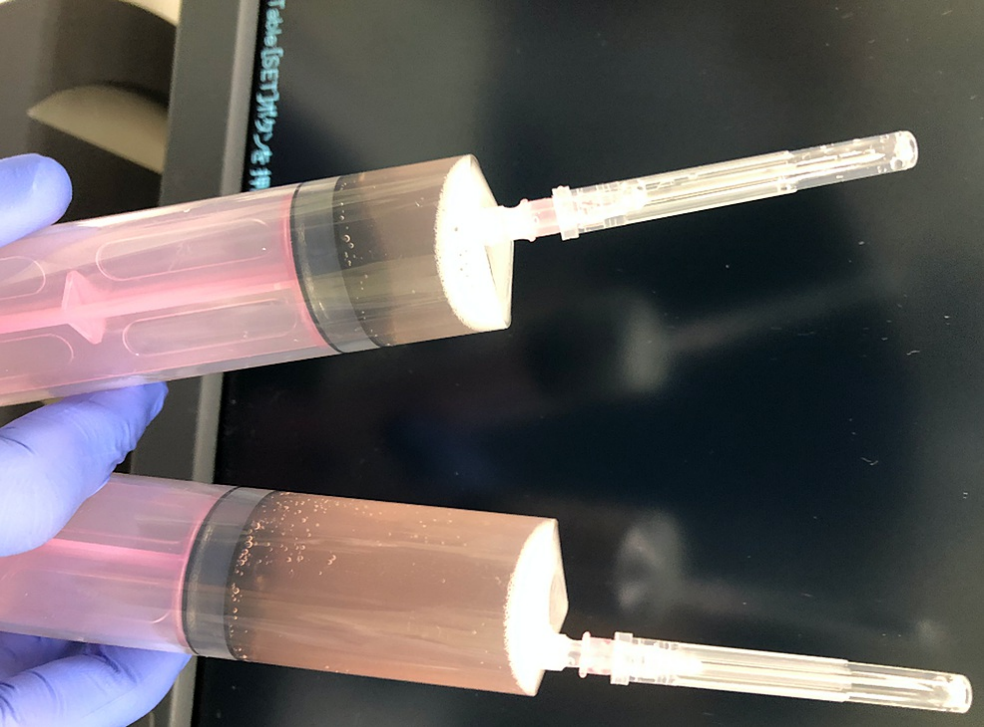
Appearance of bronchoalveolar lavage fluid from the patient. Bronchoalveolar lavage fluid (BALF) was collected from right B3 with 44/100 mL of recovery. The BALF was slightly reddish white colored and cloudy. The upper syringe is the first one, and the lower syringe is the second one.

Bronchoscopy was performed on the day of admission. Bronchoalveolar lavage fluid (BALF) was collected from right B3 with 44/100 mL of recovery. The BALF was slightly reddish white colored and cloudy (Figure 3). Examination of the BALF revealed the following: 1,801 /µL total cell count with lymphocytosis (53% of lymphocytes, 43% of histiocytes/monocytes, and 4% of neutrophils), a cluster of differentiation (CD)4/CD8 T cell ratio of 2.31, and negative cultures for bacteria and fungi. A PCR test for *Pneumocystis jirovecii* in BALF was negative. Cytology was also negative for pathogens and malignant cells.

Based on the clinical course, chest image findings, and BALF results, the patient was diagnosed with drug-induced pneumonitis, possibly caused by EC therapy. Intravenous methylprednisolone (mPSL) treatment (250 mg/day for three days, followed by 125 mg/day for three days) was initiated. Symptoms, including respiratory distress and dry cough, serum levels of CRP, and chest X-ray findings, improved rapidly (Figure 1B). Corticosteroids were tapered to 30 mg/day prednisolone (PSL) for five days. She was discharged on day 13 with PSL 20 mg/day, and PSL has been successfully tapered to 5 mg/day 40 days after discharge. The patient underwent definitive surgery for breast cancer afterward.

## Discussion

We presented a case of drug-induced ILD caused by EC therapy before breast cancer surgery. As described above, drug-induced ILD was diagnosed on the basis of clinical, physiological, and radiological findings consistent with ILD, a temporal relationship between onset of symptoms and suspected drug exposure, absence of other possible causes, e.g., infection, pulmonary edema, and progression of underlying disease, and improvement upon withdrawal of the suspected causative agent with or without corticosteroid therapy [4]. In this case, we did not measure anti-*Trichosporon asahii* antibodies, other autoantibodies rather than ANCA, nor other viral antigens except SARS-CoV2. Therefore, we cannot completely exclude other causes of interstitial pneumonia including connective-tissue disease (CTD)-ILD, summer-type hypersensitivity pneumonitis, and viral infections as a limitation. In general, summer-type hypersensitivity pneumonitis can be spontaneously improved by antigen isolation. This case did not improve at all after continued hospitalization at the previous hospital. There was no relapse after discharge with corticosteroid tapering. The clinical course reduces the possibility of CTD-ILD or summer-type hypersensitivity pneumonitis.

Severity is defined by an internationally accepted severity classification (Common Terminology Criteria for Adverse Events (CTCAE) version 5.0): grade 1 (mild), asymptomatic, radiographic findings only; grade 2 (moderate), symptomatic, not interfering with activities of daily living; grade 3 (severe), symptomatic, interfering with activities of daily living or oxygen indicated; grade 4 (life threatening), life threatening, or ventilator support required; and grade 5 (fatal). In the case described here, severity was considered CTCAE grade 3.

High-resolution computed tomography (HRCT) patterns related to pulmonary drug toxicity have been reported in the literature, and the most frequent ILDs patterns reported include nonspecific interstitial pneumonia (NSIP), usual interstitial pneumonia (UIP), hypersensitivity pneumonitis (HP), organizing pneumonia (OP), and diffuse alveolar damage (DAD) [5]. Although the imaging patterns are not specific, early detection and diagnosis of drug-induced ILDs is useful for the treatment of the disease. HRCT is, therefore, an essential tool for diagnosis and improvement of patient outcomes.

Zhao et al. summarized case reports of drug-induced ILDs in breast cancer patients published between 2000 and 2019 [6]. There were 39 studies of drug-induced ILDs caused by chemotherapy agents, including epirubicin, doxorubicin, pegylated liposomal doxorubicin, cyclophosphamide, paclitaxel, albumin-bound paclitaxel, gemcitabine, fluorouracil, and monoclonal antibodies [6]. There was only one case report, which was written in Japanese, of drug-induced ILD caused by EC therapy [7]. Although the paper did not include chest CT images, based upon the description in the text, CT findings probably would have been hypersensitivity pneumonitis patterns [7]. Drug-induced ILD reported in the case report resolved spontaneously with only cessation of EC therapy [7]. However, cyclophosphamide is a well-known cause of drug-induced ILD. Thus, EC therapy should be considered a cause of ILD even if there are only a few case reports of drug-induced ILD due to EC therapy.

Considering the compatible hypersensitivity pneumonitis pattern on CT findings [3], lymphocytosis in BALF, and normal levels of serum KL-6, the pathobiology of the drug-induced ILD in the present case might have resulted from an allergic reaction. The concern was the possible complication of diffuse alveolar hemorrhage since BALF appeared slightly reddish white colored and contained red blood cells. It might have been better to perform Berlin blue staining to explore hemosiderin-laden macrophages in BALF. Fortunately, rapid tapering of corticosteroid treatment did not result in a relapse of the ILD.

## Conclusions

The patient experienced drug-induced ILD caused by EC therapy in breast cancer. Treatment intensity can be decided based on past reports, severity of the disease (CTCAE grade), CT findings, and laboratory results. This case report should be helpful for physicians who are facing similar situations.
